# Incidental pancreatic cyst: still a lot of road to cover

**DOI:** 10.1590/0100-3984.2018.51.4e1

**Published:** 2018

**Authors:** Giuseppe D'Ippolito

**Affiliations:** 1 Adjunct Professor in the Department of Diagnostic Imaging of the Escola Paulista de Medicina da Universidade Federal de São Paulo (EPM-Unifesp), Radiologist of Laboratório Fleury, São Paulo, SP, Brazil

The wide dissemination of and technological advances in imaging examinations over the last two decades have given rise to a new entity^(1)^: the incidentaloma. An incidentaloma consists of an image observed incidentally or accidentally in a patient who does not present any related symptoms or biochemical abnormalities^(1)^. Incidentalomas were first mentioned in articles published in the 1990s^(2)^. One study of the subject was published in an important medical journal in 1992^3)^. The authors addressed the cascade of examinations and interventions resulting from an incidental finding, as well as the cost of that approach and the sometimes unnecessary exposure of the patient to risks or anxiety^(3)^. In the abdominal field, hepatic, adrenal, renal, and pancreatic incidentalomas are fairly common, to the point of motivating the American College of Radiology (ACR) to create a committee to establish guidelines for the management of such findings^(4)^. In the last ten years, several international societies have published similar or sometimes contradictory guidelines or recommendations for the management of incidental pancreatic cysts^(5-7)^.

In the first studies of the topic, incidental pancreatic cysts were reportedly found in 13.0-19.5% of abdominal magnetic resonance imaging (MRI) studies^(8,9)^ and in 2.5% of computed tomography (CT) scans^(10)^. In other studies, the reported prevalence has been as low as 2.0% and as high as 45.0%^(11,12)^, indicating that although pancreatic incidentalomas might be common, their exact prevalence is still poorly understood and depends not only on the imaging method adopted and the profile of the population studied (the prevalence increasing with age) but also on variables such as the examination technique, as well as the experience and diligence of the observers.

In this issue of **Radiologia Brasileira**, Falqueto et al.^(13)^ retrospectively evaluated 924 sequential patients submitted to MRI (n = 443) or CT (n = 481) and found pancreatic cysts in 6.1% and 3.1% of the examinations, respectively, the mean prevalence being 4.5%. Among the 42 patients with pancreatic cysts, the prevalence was 17 times greater in those with pancreatic complaints (42.9%) than in those with other symptoms (2.4%). That is quite similar to the 2.5% prevalence observed in a large sample of adult patients in the United States^(12)^.

On the basis of the clinical data and imaging aspects, Falqueto et al.^(13)^ classified pancreatic cystic lesions as neoplastic or non-neoplastic. The authors found signs of malignancy risk in 26.3% of the 38 cases classified as neoplastic, the cysts being interpreted as intraductal papillary mucinous neoplasms (IPMNs) in 74% of those cases. The worrisome signs for malignancy of a cyst were a diameter greater than 30 mm, a solid component, and dilation of the main pancreatic duct. Despite the discrepancies among the various guidelines and recommendations published, there is a tendency to consider the following aspects as suspicious for malignancy in an IPMN^(5)^: a diameter ≥ 30 mm; wall thickening; no contrast enhancement of the nodule; and a main pancreatic duct diameter of 5-9 mm. The aspects considered to indicate a high risk for IPMN malignancy are an enhancing solid component within a cyst, a main pancreatic duct diameter > 9 mm without other cause of obstruction, and biliary tract dilation or jaundice secondary to a pancreatic head cyst^(5)^. Other risk factors include IPMN growth of more than 2 mm per year and an elevated level of Ca 19-9^(6)^. When one or more of these high-risk features are identified on CT or MRI scans, surgical intervention, preceded or not by endoscopic ultrasound-guided fine needle aspiration (EUS-FNA), can be considered^(5,14,15)^. Some authors recommend that any cyst that is a candidate for resection should undergo to EUS-FNA, in order to reduce the number of unnecessary surgeries^(15)^.

Among the current guidelines, there are several discrepancies in the follow-up algorithm, mainly regarding the cut-off diameter adopted to indicate a change in practice, the time between examinations, and the duration of follow-up. In a recent article published by the ACR Incidental Findings Committee, as the first revision of the document released in 2010, Megibow et al.^(15)^ stated that the recommendations are based not only on the level of evidence but also on personal experience, when evidence is lacking, and their consensus is a guidance rather than formal guidelines. The main changes proposed in the management of patients with incidental pancreatic cysts were a longer follow-up period (10 years)-due to reports of the development of invasive pancreatic adenocarcinoma after 5 years of follow-up^(16,17)^-a more liberal use of EUS-FNA, and the recommendation to discontinue follow-up beyond 80 years of age, except when there are symptoms. It should be keep in mind that only cysts with a diameter > 1.5 cm contain a sufficient volume of fluid (1-2 mL) to allow reliable cytological and biochemical analyses^(18)^. Other authors have asserted that the diagnostic accuracy of fine-needle aspiration is increased when the results are considered in association with clinical and imaging aspects^(19)^.

Another recently introduced question concerns the growth of a pancreatic cyst over time. Megibow et al.^(15)^ suggested that growth of a cyst (sufficient to raise suspicion of malignancy) can be defined as an increase in its diameter > 100% if it has an initial diameter < 0.5 cm, > 50% if it has an initial diameter of ≥ 0.5 to < 1.5 cm, and > 20% if it has an initial diameter ≥ 1.5 cm. Of course, from a mathematical point of view, a 40% increase in a 1.4-cm cyst is more significant than is a 25% increase in a 1.5-cm cyst, which makes the use of common sense essential when following this particular criteria.

In some of the guidelines adopted by various study groups, there is no concern of identifying, in cysts smaller than 2.0 or 3.0 cm, communication with the pancreatic duct, which would indicate a diagnosis of IPMN^(4,6)^. However, Megibow et al.^(15)^ proposed a specific algorithm for small cysts (1.5-2.5 cm) suspected of being IPMNs, recommending longer intervals between imaging studies for patients with cysts < 2.0 cm.

Despite the high prevalence of pancreatic incidentalomas, it is undeniable that little is known about their natural history. For example, the reported rates of cyst malignancy by morphological type vary widely in the literature, ranging from 12% to 47% for branch-duct (type II) IPMNs and from 38% to 68% for main-duct (type I) or mixed (type III) IPMNs^(20)^. That variation might be due to the histological criteria adopted to consider the malignancy of the lesion. Ultimately, it can be said that exact rates of malignancy of small incidental pancreatic cysts remain unknown^(15,21)^.

The various published guidelines adopt different cyst diameter cut-off points, ranging from 1.5 cm to 3.0 cm, to indicate changes in the timing of follow-up evaluations and needle aspiration. Therefore, once a pancreatic cyst has been identified and a pseudocyst has been excluded (on the basis of the clinical history), which of the various guidelines should be followed? For now, there is no clear answer to that question^(22)^.

In summary, there are certain relevant data of which radiologists should be aware. There is a tendency for CT and MRI, with dedicated protocols for the study of the pancreatic region, to be considered equivalent for detecting the main warning signs or risk of malignancy in the incidental pancreatic cyst^(23,24)^. Magnetic resonance cholangiography is the most effective noninvasive method for demonstrating communication between a cyst and the pancreatic duct^(1)^. Cysts < 2.0 cm are difficult to characterize, which, according to some guidelines, does not affect the management proposed, such cysts basically being monitored (with varying frequency) by imaging methods, preferably MRI. The use of abdominal ultrasound to monitor these cysts, although desirable, is still controversial because of its questionable reproducibility^(25,26)^. Indeterminate cysts are considered to be IPMNs until proven otherwise, because of their high prevalence^(15)^. Serous cystadenoma and pancreatic pseudocyst can be diagnosed on the basis of their typical radiological features and of the history of pancreatitis, respectively. Small pancreatic cysts need not be followed up in asymptomatic patients who remain stable for 10 years. In patients over 80 years of age, the need for the monitoring of pancreatic cysts in asymptomatic patients is highly debatable. There is a tendency for some radiologists do not report pancreatic cysts smaller than 5 mm (known as "white dot" cysts) found incidentally in patients over 75 years of age^(15)^. In patients with pancreatic cysts that show growth or other warning signs for malignancy, there has been an increase in the use of EUS combined with FNA, in order to measure the carcinoembryonic antigen level, the amylase level, and to identify the presence of mucin^(18)^.
